# The Analysis of the Frequency of Leukoplakia in Reference of Tobacco Smoking among Northern Polish Population

**DOI:** 10.3390/ijerph17186919

**Published:** 2020-09-22

**Authors:** Aida Kusiak, Adrian Maj, Dominika Cichońska, Barbara Kochańska, Aleksandra Cydejko, Dariusz Świetlik

**Affiliations:** 1 Department of Periodontology and Oral Mucosa Diseases, Medical University of Gdańsk, 80-204 Gdańsk, Poland; adrianm@gumed.edu.pl (A.M.); dominika.cichonska@gumed.edu.pl (D.C.); aleksandra.cydejko@gumed.edu.pl (A.C.); 2 Department of Conservative Dentistry, Medical University of Gdańsk, 80-208 Gdańsk, Poland; bkochan@gumed.edu.pl; 3 Department of Biostatistics and Neural Networks, Medical University of Gdańsk, 80-211 Gdańsk, Poland; dswietlik@gumed.edu.pl

**Keywords:** precancerous lesions, leukoplakia

## Abstract

*Objective*: The aim of the study was an updated analysis of the frequency of leukoplakia in reference to tobacco smoking among the northern Polish population. *Material and Methods:* Medical records of 5720 patients who suffer from abnormalities and oral mucosa diseases between January 2015–December 2018 were analyzed. Among them, 416 medical charts of patients with leukoplakia were selected. The study group consisted of 196 women and 220 men aged between 21–86 years (average 45.6 years). The analysis was conducted in terms of age, gender, and smoking tobacco. The basic criterion for inclusion in the study was the presence of oral leukoplakia confirmed by histopathological examination, recorded in the chart. Information about the patient’s active smoking was obtained from documented medical interviews. An active smoker was defined as a patient who smoked 10 or more cigarettes a day for at least the previous six months. The study used parametric and non-parametric statistical methods. *Results:* The highest incidence of leukoplakia was found in the age group 41–60 (46.6%), where the vast majority were active smokers (85.1%), and mostly men (86.2%). However, among patients with leukoplakia, the highest prevalence of smoking was found in the age group 21–40 years (86.8%) in women, where out of 38 patients with leukoplakia, 33 were active smokers. More patients with leukoplakia were observed in groups of smokers and it was statistically significant. Homogeneous form was the most commonly diagnosed form of leukoplakia in our study; it was found in almost 95% of cases. Leukoplakia was mainly observed on the cheeks. Changes on the gums, the alveolar process or the bottom of the tongue and mouth were rarely found. *Conclusion:* Our studies revealed that there is a statistically significant correlation between tobacco smoking and the presence of oral leukoplakia among the northern Polish population. It should be noted that dentists, in particular, are capable of early diagnosis and implementation of appropriate treatment of leukoplakia and, most often, crucial elimination of the main risk factor, which is smoking, and the implementation of effective tobacco control interventions.

## 1. Introduction

All lesions, the occurrence of which increases the risk of malignant transformation, are called precancerous states; this is a typical clinical concept. Although the etiology of oral leukoplakia has not yet been fully defined, there are numerous local risk factors of precancerous lesions, which may significantly increase the risk of malignant transformation. Undoubtedly, there should be mentioned tobacco, excess alcohol use, chronic and mechanical trauma, and others [1,2,3,4,5].

Leukoplakia called “white spot” is a type of white keratosis, a precancerous change associated with the formation on the surface of the oral mucosa. In 1977, the World Health Organization defined the term leukoplakia as any white patch on the oral mucosa that cannot be scraped off, which is not associated with any physical or chemical factor except smoking, and that cannot be characterized as any other definable lesion or disease [1,2,3,5].

Currently, the most commonly used classification is that of van der Waal, which has the broadest clinical application [4]. This classification takes into account the size changes of leukoplakia, but also the clinical picture (homogeneous and non–homogeneous), as well as changes in the histopathological examination. Such distribution allows for the separation of the four stages of advancement of the disease. Each subsequent (higher) level of leukoplakia in the classification of van der Waal is associated with an increased risk of malignant transformation of cancer [4,5,6,7].

The basis for the formation of leukoplakia is a disordered process of epithelial keratosis. Clinical changes in the nature of leukoplakia may be more or less marked out, of various sizes. Color may be changed from a slightly off-white to highly intense white. Homogeneous leukoplakia, which is most commonly observed clinically, has a smooth and flat surface with well-demarcated borders and exhibits shallow cracks of the surface keratin. Non- homogeneous leukoplakia is classified into three clinical categories, which are speckled leukoplakia, nodular leukoplakia, and verrucous leukoplakia [5,6]. The distinction between homogenous and non-homogenous leukoplakia is based only on the clinical picture, mostly on the color of the lesion surface and morphological features as thickness. Therefore, this distinction is of great significance due to the fact that it does have some bearing on the outcome or prognosis. Homogeneous lesions are characterized by a relatively low risk of malignant transformation. However, non-homogeneous leukoplakia is the one which usually carries a much higher risk of malignant transformation [4,5,6].

Leukoplakia is only a clinical term and the lesions are not characterized with any specific histology. Histopathology examination may reveal either atrophy or hyperplasia (acanthosis). Although epithelial dysplasia might be observed, it is not present in every case of oral leukoplakia. The presence of epithelial dysplasia is the strongest factor of possibility in future malignant transformation in any oral potential malignant disorder, including leukoplakia [6].

Oral leukoplakia might be characterized by various clinical pictures, which can create difficulties with a proper diagnosis. Moreover, there are several disorders that must be excluded before the diagnosis of oral leukoplakia, like acute pseudomembranosus candidosis, discoid lupus erythematosus, leukoedema, lichen planus, lichenoid reaction, frictional keratosis, chemical injury, hairy leukoplakia and leukokeratosis nicotina palate. Only in the case of lichen planus lichenoid reaction and discoid lupus erythematosus are biopsy and histopathology required to distinguish those diseases from oral leukoplakia. In the case of suspicion of an acute pseudomembranosus candidosis, a swab for culture is sufficient examination. The other diseases mentioned require only an insightful medical interview that includes drug history, habitual cheek-lip biting, or other pathological habits, and a proper clinical examination of oral mucosa [6,7].

The most common cause of leukoplakia should be smoking (in the form of cigarettes and pipes), alcohol, or long-term mechanical or thermal trauma of oral mucosa. Some authors recognize human papillomavirus infection as the risk of changes in the type of white keratosis. The most common locations of changes are mucosal cheek, lower lip and palate. A proper diagnosis is made by histopathology biopsy taken from clinically diagnosed white changes, which is crucial for an appropriate treatment and determination of the risk for malignant transformation. Leukoplakia belongs to a group of competent precancerous diseases, where the probability of malignancy is 10–20%, so the average is determined [4,5,7]. However, development of leukoplakia might be affected by subject characteristics such as age, gender, or even a geographical area of residence. According to a systematic review prepared by Petti et al., the global frequency of oral leukoplakia is between 1.7% and 2.7%. There are no significant differences in frequency of oral leukoplakia between geographical areas, which might be caused by current similar tobacco usage and alcohol consumption in Europe, North America and South Asia, which are the main risks of development of this oral mucosa lesion. However there are differences between the distribution of oral leukoplakia among male and female patients. In developing countries, the difference between genders is higher than in developed countries [7]. According to research conducted in Poland by Petkowicz et al., the prevalence of oral leukoplakia is 11.5% of all oral mucosal diseases [4]. There are no current data concerning the frequency of occurrence of oral leukoplakia in Poland. The aim of our study was to estimate the frequency of oral leukoplakia in reference tobacco smoking in the Polish population. This study was conducted in the Department of Periodontology and Oral Mucosal Diseases of the Medical University of Gdańsk, which is a reference center for oral mucosal diseases for northern Poland. Although all patients who participated in this study came from a specific part of Poland, they might be perceived as a representative group of the entire Polish population, due to the slight differentiation of development of our country.

## 2. Materials and Methods

Medical records of 5720 patients of the Department of Periodontology and Oral Mucosa Diseases of the Medical University of Gdansk, all of whom suffer from oral mucosa diseases and abnormalities between January 2015–December 2018, were analyzed. Among them, 416 medical charts of patients with clinically observed and histopathologically confirmed leukoplakia were selected. The study group consisted of 196 women and 220 men between 21–86 years of age (average 45.6 years). The analysis was conducted in terms of age, gender, and smoking tobacco. The basic criterion for inclusion in the study was the presence of oral leukoplakia, confirmed by histopathological examination, recorded in the chart. Information about the patient’s active smoking was obtained from documented medical interviews. An active smoker was defined as a patient who smoked 10 or more cigarettes a day for at least the previous 6 months.

The agreement for research No. NKEBN/266/2011 was given by the Bioethics Committee of Medical University of Gdansk.

### Statistical Methods

The statistical analyses have been performed using TIBCO Software Inc. (2017), statistica (TIBCO Software, Palo Alto, CA, USA), version 13, http://statistica.io. The quantitative variables were characterized by the arithmetic mean of the standard deviation or median or max/min (range) and 95% confidence intervals. The qualitative variables are presented as counts and percentages. The W. Shapiro–Wilk test was used to check if the quantitative variable comes from a normally distributed population. On the other hand, the Leven (Brown–Forsythe) test was used to prove the homogeneity of variance hypotheses, and the statistical significance of discrepancies between the two groups was processed using the Student’s t-test or the Mann–Whitney U test. Chi-square tests of independence were used for qualitative variables. In order to determine the relationship, strength and direction between the variables, the correlation analysis was used by determining the Pearson correlation coefficient (and the Spearman’s rank order). In all calculations the level of statistical significance was assumed at *p* < 0.05.

## 3. Results

Table 1 shows the percentage of patients with leukoplakia, by gender and age and smoking. This type of oral mucosal lesion was found in 416 patients (196 women and 220 men), of which 363 patients (160 women and 188 men) smoked cigarettes. Thus, the percentage of smokers with leukoplakia was a total of 83.2% (82.9% in women and 83.5% among men). In the 21–40 age group, leukoplakia was found in 63 people who smoked cigarettes, which constituted 86.3% of the respondents, while in the non-smoking group, leukoplakia occurred in 10 people, which constituted 13.7% of the respondents. In the 41–60 age group, 165 patients (women and men) who smoked had leukoplakia, which constituted 85.1% of the respondents, while in non-smokers, leukoplakia was found in 29 people, which constituted 14.9% of the study participants. In the group of patients over 60 years of age, leukoplakia was found in 123 people, who smoked cigarettes, which constituted 82.6% of the respondents; however, in the group of non-smokers, it was found in 26 people, which constituted 17.4% of patients examined. Statistical analysis showed statistically significant differences in the group of non-smokers and smokers in each age group (*p* < 0.05). Analyzing the group of women, it was found that in the 21–40 age group, leukoplakia occurred in 33 female subjects who smoked cigarettes, which constituted 86.8% of female study participants in this age category, while in the group of non-smokers, leukoplakia occurred in 5 women, which constituted 13.2% of the examined women in this particular age category. In the 41–60 age group, 80 women participating in the study who smoked had leukoplakia, which constituted 83.3% of female patients in this age category, while in non-smokers, leukoplakia was found in 16 examined women, which constituted 16.7% of the female respondents aged 41–60 years old. Furthermore, in the group of female patients over 60 years of age, leukoplakia was found in 50 women, which constituted 83.8% of female subjects in this age category; however, in the non-smoking group, leukoplakia was found in 12 women, which constituted 16.2% of female study participants aged over 60 years old. In the entire group of women, leukoplakia was found in 160 female patients, which constituted 82.9% of all women participating in the study, while in the non-smoking group, leukoplakia was found in 36 women examined, which constituted 17.1% of all female participants. Statistical analysis showed statistically significant differences in the female group of non-smokers and smokers in each age group (*p* < 0.05).

On the over hand, analyzing the group of men, it was found that in the 21–40 age group leukoplakia occurred in 30 people who smoked cigarettes, which constituted 85.7% of the male respondents in this age category, while in the group of non-smokers, leukoplakia occurred in 5 participants of the study, which constituted 14.3% of men aged 21–40 years old. In the 41–60 age group, 85 men who smoked had leukoplakia, which constituted 86.2% of male patients participated in the study in this particular age range, while in non-smokers, leukoplakia was found in 13 men, which constituted 17.8% of the male respondents aged 41–60 years old. Furthermore, in the group of male patients over 60 years of age, leukoplakia was found in 73 study participants, which was 83.9% of the respondents in this age category, however, in the non-smoking group, leukoplakia was found in 14 men, which accounted for 16.1% of male patients aged over 60 years old. In the entire group of men, leukoplakia was found in 188 people, which constituted 83.5% of all male study participants, while in the non-smoking group, leukoplakia was found in 32 people, which constituted 16.5% of the male respondents. Statistical analysis showed statistically significant differences in the male group of non-smokers and smokers in each age group (*p* < 0.05).

**Figure 1:**
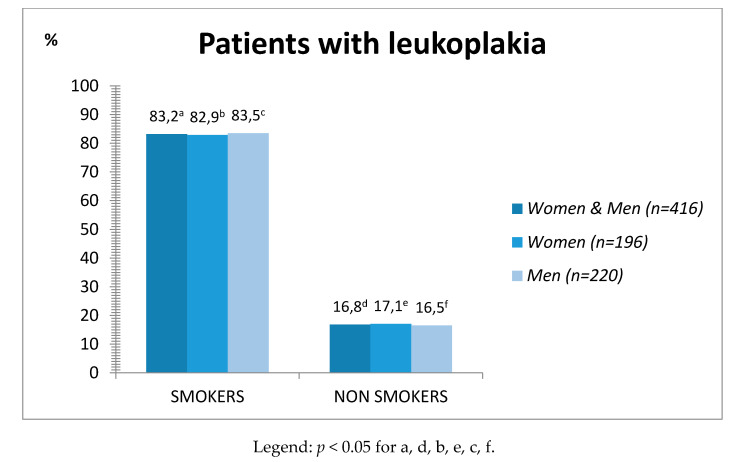
Percentage of patients diagnosed with leukoplakia smokers and non-smokers.

The highest incidence of leukoplakia was found in the age group 41–60 (46.6%), where the vast majority were active smokers (85.1%) and mostly men (86.2%). However, among patients with leukoplakia the highest prevalence of smoking was found in the age group 21–40 years (86.8%) in female patients. More patients with leukoplakia were observed in groups of smokers, and this was statistically significant (*p* < 0.05) (Figure 1).

Homogeneous form was the most commonly diagnosed form of leukoplakia in our study, it was found in almost 95% of cases. Leukoplakia was mainly observed on the cheeks. Changes in the gums, the alveolar process or the bottom of the tongue and mouth were rarely found.

## 4. Discussion

Given the etiology of leukoplakia, many authors have demonstrated the synergistic effect of tobacco and alcohol [1,3,8,9,10,11,12,13,14]. The most important factor is the time of the impact of these risk factors [3,8,11,12,13,15].

The study showed the presence of leukoplakia in 416 cases (6.1%) of a total of 5720 patients with diseases of the oral mucosa, as observed at a similar level by other researchers [1,16,17]. Epidemiological data from analyzing the frequency of leukoplakia ranged from 0.2 to 11.7% [2,4,6,18,19,20,21,22,23,24,25,26]. In our study, we observed a slight downward trend in the occurrence of leukoplakia. We also noted the highest frequency of leukoplakia in the middle age group, consisting of patients aged 41–60 years old (46.6%), with a slight predominance of men (98 men to 96 women with lesions). Mostly higher incidence of leukoplakia, in men aged 41–60 years, has also appeared in the work of some authors [8,18,25], although some investigators have shown that the incidence of leukoplakia in women and men is equal [4], or even higher in women [17]. According to Wang et al., in a retrospective study including 875 patients from a Chinese population, women more often had oral leukoplakia (the ratio of men to women for oral leukoplakia was 1:2) and the malignant transformation rate in women (14.4%) was higher than in men (9.4%) [27]. In our retrospective study, leukoplakia was diagnosed slightly more often in men, but women accounted for 47.1% of all diagnosed patients (220 men and 196 women with leukoplakia lesions). According to Wang et al., another factor, which was also significant in the prevalence and malignant transformation of oral leukoplakia, was the age of the patients. Looking at the results that they obtain, oral leukoplakia was more common in the elderly (65.7% of patients were over 50 years of age). The authors also observed that malignant transformation was mostly appearing among patients aged 50–59 (27.8%) and 60–69 (25%). In this group of patients, only 18.9% were smokers and 12.9% consumed alcohol. However, the malignant transformation rate of oral leukoplakia among currently smoking patients was lower (3%) than among non-smokers or former smokers (14.4%) and there were no significant differences in malignant progression between women and men [27]. In our study, the highest incidence of leukoplakia was found in the age group 41–60, which constituted 46.6% of patients diagnosed with leukoplakia, where a slight advantage was noted in men (98 men to 96 women). However, people over 60 years of age also constituted the second most common group of leukoplakia (35.8% of patients with leukoplakia). In both of these age categories, smokers accounted for the overwhelming majority (over 80% in both women and men, with a slight majority in men). Jayasooriya et al., similarly to our results, also found men to be more affected by leukoplakia: in a group of 93 cases of leukoplakia, men predominated with a ratio of men to women of 3.5: 1.That being said, this study revealed that there was no statistically significant difference in the frequency of malignant transformation of oral leukoplakia connected with gender. In this study the age of patients also did not influence the risk of malignant transformation [28]. On the other hand, in retrospective research conducted by Rubert et al., in a group of 412 patients diagnosed with oral leukoplakia, females (281) predominated over males (131) [29]. In that research most patients (53.2%) were also non-smokers and did not consume alcohol (85%). In 81.6% of cases, homogeneous leukoplakia was diagnosed and only 8.3% of cases presented malignant progression of oral leukoplakia. However, the authors observed an interesting association between patients with no history of cigarette smoking and the presence of clinically non-homogeneous forms of oral leukoplakia. In analysis of the data from our research, we observe a strong relationship between the occurrence of oral leukoplakia and cigarette smoking, and the most commonly diagnosed form of leukoplakia was the homogeneous form. Moreover, a study by Rubert et al. provides us with information that gender may also influence the risk of malignant transformation of oral leukoplakia. In the study, 57.1% of cases were diagnosed among female patients and 42.9% among male patients, which may lead to a conclusion that female patients might be more prone to malignant transformation of oral leukoplakia [29], which is the opposite result of the research conducted by Jayasooriya et al. [28], Wang et al. [27], and meta-analysis prepared by Matulić et al., which also revealed that there were no statistically significant differences in malignant transformation of oral leukoplakia between male and female patients [30]. Nevertheless, returning to the statement that the age of patients may influence the clinical symptoms of leukoplakia, the study by Rupert et al. also seems to confirm this [29]. In their research, patients with clinically non-homogeneous lesions were older and lesions of non-homogeneous leukoplakia were larger than clinically homogenous to a statistically significant degree. In addition, Mello et al., in their systematic review, presented most studies stating that oral leukoplakia and other oral potentially malignant disorders more often affect patients aged over 50 [31]. Based on the available data, the authors estimated that the total incidence of potentially malignant oral diseases, including leukoplakia, was 4.47%.

A high percentage of patients with leukoplakia in the age groups above 50 years of age was also reported by other researchers [4,17,32]. A meta-analysis prepared by Pinto et al. revealed that the majority of analyzed studies confirm a higher prevalence of oral leukoplakia malignant transformation among older patients, and that there were no statistically significant differences in malignant transformation of oral leukoplakia between male and female patients, however the odds ratio favored male patients. This analysis included seventeen studies describing malignant transformation of oral leukoplakia according to gender. Overall mean proportion of malignant transformation of oral leukoplakia in this study was estimated to be 9.7% [32]. In our study, older patients over 60 years of age also constituted a significant group of patients with leukoplakia. The majority of this group was dominated by men, who constituted 20.9% (87) of patients with lesions. The vast majority of them were active smokers, who constituted 83.9% (73) of patients in this group.

Worth noticing is the fact that in our research only 47.7% of women and 42.5% of men diagnosed with leukoplakia consciously reported this problem to a dentist. In the remaining patients, leukoplakia was detected accidentally. Active smokers constituted an overwhelming majority, both in the group of men and women and in each age category at over 80%. Surprisingly the highest prevalence of smoking was found in the youngest age group, 21–40 years old (86.8%), in women. Tobacco smoking is an important factor in the pathogenesis of leukoplakia, as many researchers have indicated in their studies [2,8,10,24,33]. In the retrospective study conducted by Chaturvedi et al., in a group of 4886 patients with oral leukoplakia, 42.5% had a history of smoking and 5.2% of alcohol abuse. Leukoplakia was most common among patients aged 50–59. The risk of oral cancer among patients with diagnosed oral leukoplakia was estimated at 3.3% [34].

In our study, the most commonly diagnosed form of leukoplakia was the homogeneous form, which was diagnosed on the cheeks, less often on the gums and tongue. Although, according to Brouns et al., there is no association between localization of oral leukoplakia lesions and risk of malignant transformation [35], Rubert et al. still observed that lesions localized on the tongue present a higher risk of malignization than other localizations of leukoplakia in the oral cavity [29]. Furthermore, systematic review conducted by Pinto et al. revealed that leukoplakia lesions localized on both tongue and flor of the mouth present a higher risk of malignant transformation, however the tongue still remains the most common localization for the malignant transformation of oral leukoplakia [32].

A limitation in our research was, in particular, its retrospective nature, therefore some information on risk factors, i.e., alcohol consumption, mechanical trauma, and eating habits of patients were unavailable to us, so it was not possible to reliably assess them in this study.

## 5. Conclusions

Our study revealed that there is a statistically significant correlation between tobacco smoking and the presence of oral leukoplakia among the northern Polish population. It should be noted that dentists, in particular, are capable of early diagnosis and implementation of appropriate treatment of leukoplakia and, most often, crucial elimination of the main risk factor, which is smoking, and the implementation of effective tobacco control interventions.

## Figures and Tables

**Table 1:** Number and percentage of patients diagnosed with leukoplakia smokers and non-smokers by gender and age.

Age (in Years)	Patients with Leukoplakia				
	Total Number of Patients	Smokers		Non-Smokers	
	*N (%)*	*n*	*%*	*N*	*%*
**Women and men**					
**21–40**	73 (17.6)	63 ^a^	*86.3*	10 ^b^	*13.7*
**41–60**	194 (46.6)	165 ^c^	*85.1*	29 ^d^	*14.9*
**>60**	149 (35.8)	123 ^e^	*82.6*	26 ^f^	*17.4*
**Total**	**416**	**363 ^g^**	***83.2***	**53 ^h^**	***16.8***
**Women**					
**21–40**	38 (9.1)	33 ^i^	*86.8*	5 ^j^	*13.2*
**41–60**	96 (23.1)	80 ^k^	*83.3*	16 ^l^	*19.7*
**>60**	62 (14.9)	50 ^m^	*83.8*	12 ^n^	*16.2*
**Total**	**196 (47.1)**	**160 ^o^**	***82.9***	**36 ^p^**	***17.1***
**Men**					
**21–40**	35 (8.4)	30 ^r^	*85.7*	5 ^s^	*14.3*
**41–60**	98 (23.6)	85 ^t^	*86.2*	13 ^u^	*17.8*
**>60**	87 (20.9)	73 ^w^	*83.9*	14 ^y^	*16.1*
**Total**	**220 (52.9)**	**188 ^x^**	***83.5***	**32 ^z^**	***16.5***

*p* < 0.05 for a, b, c, d, e, f, g, h, i, j, k, l, m, n, o, p, r, s, t, u, w, y, x, z.
